# A Stereomicroscopic Evaluation of Dentinal Cracks at Different Instrumentation Lengths by Using Different Rotary Files (ProTaper Universal, ProTaper Next, and HyFlex CM): An Ex Vivo Study

**DOI:** 10.1155/2016/8379865

**Published:** 2016-06-29

**Authors:** Fauzia Ashraf, Pushpa Shankarappa, Abhinav Misra, Asheesh Sawhney, Nandamuri Sridevi, Anu Singh

**Affiliations:** Department of Conservative Dentistry and Endodontics, Rama Dental College, Hospital and Research Centre, Lakhanpur, Kanpur 208024, India

## Abstract

*Introduction*. The aim of the present study was to evaluate the dentinal cracks after root canal preparation with rotary files: Gates Glidden, ProTaper Universal, ProTaper Next, and HyFlex CM at different instrumentation lengths.* Methodology*. Sixty-five mandibular premolars were mounted in the acrylic tube with simulated periodontal ligaments and the apex was exposed. The root canals were instrumented with different rotary files, namely, ProTaper Universal, ProTaper Next, and HyFlex CM, to the major apical foramen (AF), short AF, and beyond AF. The root apex was stained with 1% methylene blue dye and digital images of apical surface of every tooth were taken and development of dentinal defects was determined by using stereomicroscope. Multinomial logistic regression test was performed to identify influencing factors.* Results*. Instrumentation with rotary files terminated 2 mm short AF and did not cause any cracks. Significantly less cracks were seen when instrumentation with rotary files terminated 1 mm short apical foramen when compared with the instrumentation terminated at or beyond apical foramen (*p* < 0.05).* Conclusion*. ProTaper Universal rotary files caused more dentinal cracks than ProTaper Next and HyFlex CM. Instrumentation short AF reduced the risk of dentinal defects.

## 1. Introduction

Endodontic diseases are associated with multiple bacterial infections, where root canal therapy serves as a significantly effective treatment modality [1]. Canal preparation is one of the most important factors in successful root canal treatment, where the apical enlargement ensures cleanliness and improves the quality of canal filling [2]. A long series of instruments from stainless steel hand files and several rotary drills to integrating Nickel-Titanium (NiTi) files for shaping canals have been introduced for this purpose. The instrumentation of the root canal alone significantly weakens the roots. Stresses are generated from inside of the root canal which are higher in the apical region and more along the walls so the distribution of stress in apical area leads to development of cracks and fracture. Clinically bacteria may proliferate in crack lines and later establish biofilms on the root surface [3]. ProTaper Universal (PTU) file is made from conventional super-elastic NiTi wire and has convex triangular cross-sectional design with progressive taper over the file length and aggressive cutting action resulting in removal of relatively more dentin in a coronal way [4]. ProTaper Next (PTN) shaping files have an off-centered rectangular design and progressive and regressive percentage tapers on a single file and are made from memory wire (M-wire) technology. The off-centered rectangular design decreases the screw effect, dangerous taper lock, and torque on the file by minimizing the contact between the file and the dentin [5].

HyFlex CM (HYCM) file systems are newer type of novel NiTi systems comprising mechanical shaping files, are made of control memory wire (CM wire), do not have shape memory, utilize the heating and cooling methods to reduce cyclic fatigue, and improved safety in severely curved canals. These instruments have a symmetrical cross-sectional design with 3 cutting edges [6]. Till date, only few studies have shown that the HyFlex CM files produce the least number of dentinal cracks.

To the best of our knowledge, there is no data in the literature about the influence of these novel rotary NiTi files on the occurrence of root canal wall cracks at different instrumentation lengths. Thus, the purpose of this study was to observe the incidence of cracks in root dentin after root canal shaping procedure at different instrumentation lengths performed with the newly introduced HyFlex CM, ProTaper Next, and ProTaper Universal instruments under stereomicroscope.

## 2. Materials and Methods

### 2.1. Armamentarium Used

The following were used:

1. Sixty-five extracted human mandibular first premolar.
2. Rotary files: ProTaper Next (Dentsply Malleifer, Ballaigus, Switzerland), ProTaper Universal (Dentsply Malleifer, Ballaigus, Switzerland), and HyFlex CM (Coltene Whaledent, Altstetten, Switzerland).
3. K-file number #15 (Dentsply Malleifer, Ballaigus, Switzerland).
4. Aluminium foil (Superwrap, Hindalco, India).
5. Acrylic resin (Trulon, Triveni chemicals, Vapi, India).
6. Acrylic tube.
7. Vinyl polysiloxane impression material (Kerr, Take 1 Advanced, USA).
8. 1% methylene blue solution (Qualikems, India).
9. Endo motor X-smart (Dentsply Malleifer, Ballaigus, Switzerland).
10. 27 gauge needle and syringe (5 mL).
11. 2% sodium hypochlorite (Prevest Denpro Limited, Germany).
12. Stereomicroscope (Olympus CX41, Canada).
13. Camera (Magnus, India).

### 2.2. Methodology

A total of sixty-five human mandibular premolars extracted due to orthodontic and periodontal reasons and teeth with single straight root were selected and stored in normal saline till use. Radiographs were taken to verify single canal. The crown was removed 2 mm above proximal cement-enamel junction. The distance between the coronal and apical foramen of each root was determined by inserting a size 15 file into the canal until tip of the file was visible. Then, the root was wrapped with single layer of aluminium foil and embedded in acrylic resin set in acrylic tube. The root was then removed from tube and aluminium foil was peeled off.

Hydrophilic vinyl polysiloxane impression material was used to replace the space created by foil representing a stimulated periodontal ligament and the root was immediately repositioned. The apical 3 mm of root was exposed and immersed in water during instrumentation.

Each canal was irrigated by using a syringe and 27 gauge needle with 2% sodium hypochlorite solution between the uses of each instrument. The samples were randomly divided into three experimental groups of twenty teeth in each which were further divided into four subgroups of five teeth according to different instrument lengths and five teeth acted as a control group, which were left uninstrumented.

*Group 1*. Canal was left uninstrumented (control group).

*Group 2*. Canal was enlarged with ProTaper Next file at different instrumentation lengths in sequence of X1, X2, X3, and X4 at 300 rpm and 200 g/cm torque.

*Group 3*. Canal was enlarged with ProTaper Universal file at different instrumentation lengths in sequence of Sx, S1, S2, F1, F2, F3, and F4 at 250 rpm.

*Group 4*. Canal was enlarged with HyFlex CM file at different instrumentation lengths in sequence of 25/0.08, 25/0.06, 30/0.06, and 40/0.04 at 500 rpm and 250 g/cm torque.

 These extracted teeth were instrumented at different instrumentation lengths:

 
*AF:* instrumentation terminated at AF.
 
*AF + 1:* instrumentation terminated at 1 mm beyond the AF.
 
*AF − 1:* instrumentation terminated at 1 mm short AF.
 
*AF − 2:* instrumentation terminated at 2 mm short AF.

Root apex was horizontally sectioned 2 mm from the apical foramen with low speed seen under water cooling.

After the canal preparation and horizontal sectioning, the root apex was stained with 1% methylene blue dye and images were recorded after instrumentation by using stereomicroscope at 100x magnification for evaluation of cracks.

To define crack formation, 2 different categories were made (i.e., no cracks or cracks). Cracks were observed as stained lines extending either from the root canal lumen to the dentin or from the outer surface into the dentin were considered as cracks and the number of cracks seen on experimental tooth varied as some of the tooth showed more than one crack.

## 3. Root Canal Instrumentation Image Recording

Instrumentation with PTN, PTU, and HyFlex CM was done with a low torque motor at a contact speed of 300 rpm, 250 rpm, and 500 rpm, respectively. Composite resin was used to fix rubber stopper to control instrumentation length.

When instrumentation length was AF 1 mm or AF 2 mm, apical patency was maintained after each file by inserting #15 K-file until it appeared at AF. Each canal was irrigated by using a syringe and a 27-gauge needle with 2 mL of freshly prepared 2% NaOCl solution between the uses of each instrumentation.

ProTaper Next was used in full instrumentation lengths in the following sequence X1, X2, and X3 and the images were recorded after the apical enlargement at different instrumentation length.

ProTaper Universal file system was used in full instrumentation length in the following sequence as S1, S2, F1, F2, F3, F4, and F5 and ProTaper Universal shaping files enlarged and shaped the coronal 2nd/3rd of the canal and finishing files finish the apical third of the canal. The images were recorded after the apical enlargement at different instrumentation length.

HyFlex CM file was used in the following sequence as 08/25, 04/20, 04/25, 06/20, 04/30, and 04/40 till full instrumentation length and the images were recorded after apical enlargement.

## 4. Statistical Analysis

Multinominal logistic regression test was performed to compare the incidence of root cracks among the experimental groups. The frequency of cracks was summarized in number and percentage. Frequency of cracks among the groups was compared by chi-square (*χ* ^2^) test. A two-tailed *p* value less than 0.05 (*p* < 0.05) was considered statistically significant. Analyses were performed on SPSS software (windows version 17.0).

## 5. Results

**Figure 1:**
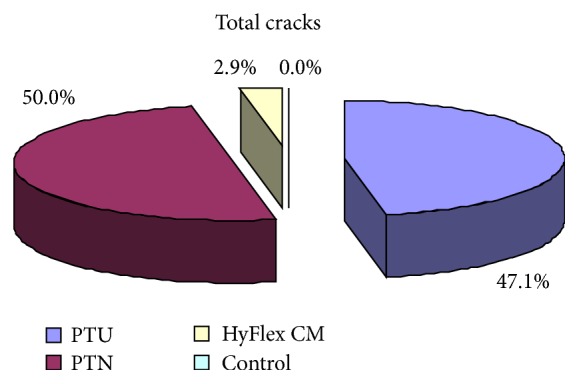
Incidence of cracks in four groups.

The frequency distribution (incidence) of apical root microcracks after canal preparation with rotary files at different instrumentation is summarized in Table 1. Of all 65 samples, total 34 cracks were found in all four groups. The maximum 17 cracks were found in PTU followed by 16 in PTN, 1 in HyFlex CM, and 0 in control group and thus the incidence of cracks is maximum in PTU (50.0%) followed by PTN (47.1%), HyFlex CM (2.9%), and control group (0.0%) (Table 1 and Figure 1).

**Figure 2:**
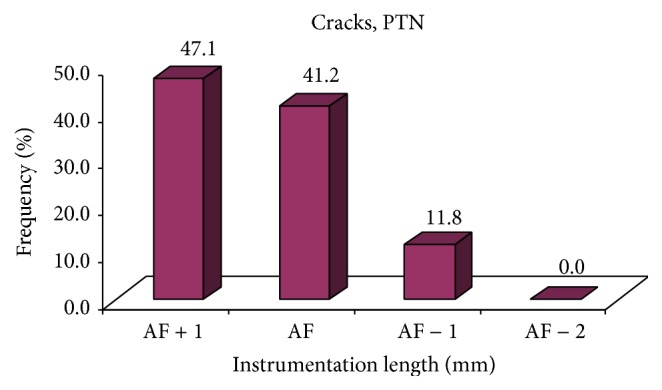
Incidence of cracks at different instrumentation length of PTN group.

Of total 16 cracks in PTN, the incidence was highest at AF + 1 mm (50.0%) followed by AF (31.3%), AF − 1 mm (18.8%), and AF − 2 mm (0.0%) (Table 1 and Figure 2).

**Figure 3:**
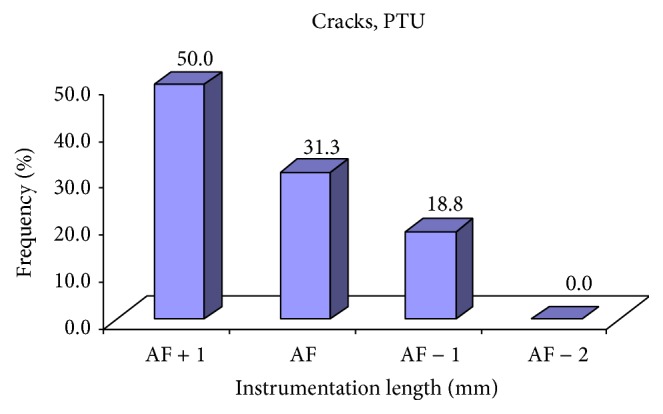
Incidence of cracks at different instrumentation length of PTU group.

Similarly, of total 17 cracks in PTU, the incidence of was highest at AF + 1 mm (47.1%) followed by AF (41.2%), AF − 1 mm (11.8%), and AF − 2 mm (0.0%) (Table 1 and Figure 3).

**Figure 4:**
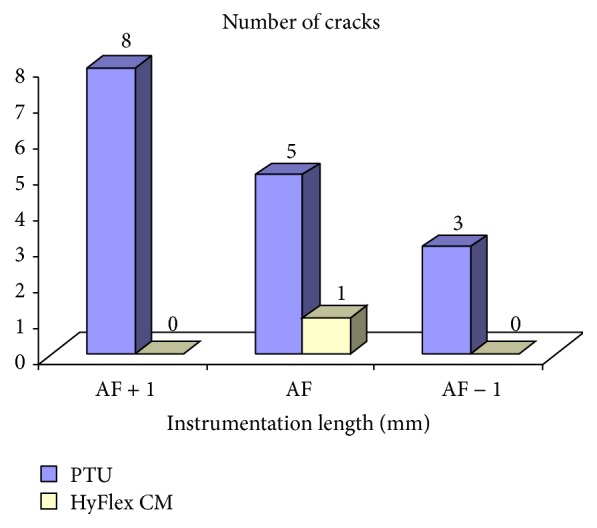
Comparison of incidence of cracks at different instrumentation length between PTU and PTN groups.

Similarly, of total 1 cracks in HyFlex CM, the cracks were evident only at AF, while they are not evident in AF + 1 mm, AF − 1 mm, and AF − 2 mm (Table 1 and Figure 4).

However, in control group, the cracks were not evident in all the instrumentation length.

**Figure 5:**
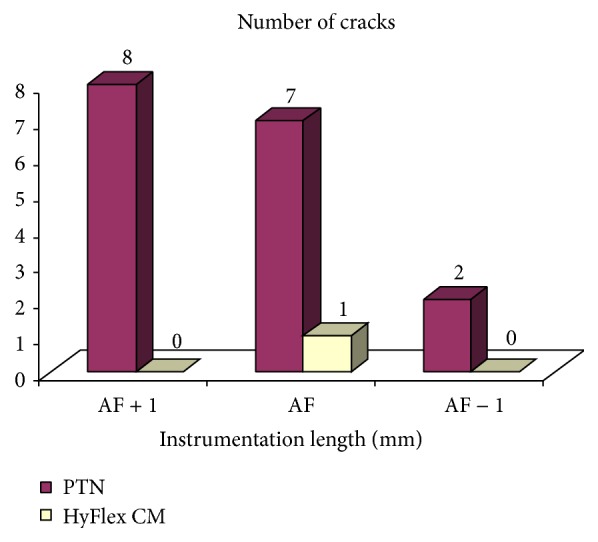
Comparison of incidence of cracks at different instrumentation length between PTN and HyFlex CM groups.

The comparison of incidence of cracks between PTU and PTN groups at comparable instrumentation length (AF + 1 mm, AF, and AF − 1 mm) is summarized in Table 2 and also depicted in Figure 5. On comparing, *χ* ^2^ test showed similar cracks at different instrumentation length between two groups (*χ* ^2^ = 0.50, *p* = 0.777), that is, not significant statistically.

Similarly, the comparison of incidence of cracks between PTU and HyFlex CM groups at comparable instrumentation length (AF + 1 mm, AF, and AF − 1 mm) is summarized in Table 3 and also depicted in Figure 5. On comparing, *χ* ^2^ test showed similar cracks at different instrumentation length between two groups (*χ* ^2^ = 1.95, *p* = 0.378), that is, also not significant statistically.

Further, the comparison of incidence of cracks between PTU and HyFlex CM groups at comparable instrumentation length (AF + 1 mm, AF, and AF − 1 mm) is summarized in Table 4. On comparing, *χ* ^2^ test showed similar cracks at different instrumentation length between two groups (*χ* ^2^ = 1.32, *p* = 0.516), that is, also not significant statistically.

Lastly, the comparison of incidence of cracks at comparable instrumentation length (AF + 1 mm, AF, and AF − 1 mm) between three groups (PTU, PTN, and HyFlex CM) is summarized in Table 5. On comparing, *χ* ^2^ test showed similar cracks at different instrumentation length between three groups (*χ* ^2^ = 2.16, *p* = 0.705), that is, also not significant statistically. In other words, the incidence of cracks was similar among the three rotary file types and instrumentation length thus can be used interchangeably.

## 6. Discussion

In this study, freshly extracted mandibular premolars were used because these teeth are probably more prone to be influenced by forces during instrumentation as a result of their smaller dimensions and thin dentinal walls. If large tapered files cannot induce cracks in mandibular premolar, it is unlikely that rotary files induce cracks in other teeth. In this study, canal preparation with rotary files was done at different instrumentation length as the canal shows sharp curvature at 2-3 mm from the apical foramen. No cracks were generated when instrumentation was terminated at AF − 2 mm, possibly because the sharp apical curvature was located within the apical 2 mm [7].

**Figure 6:**
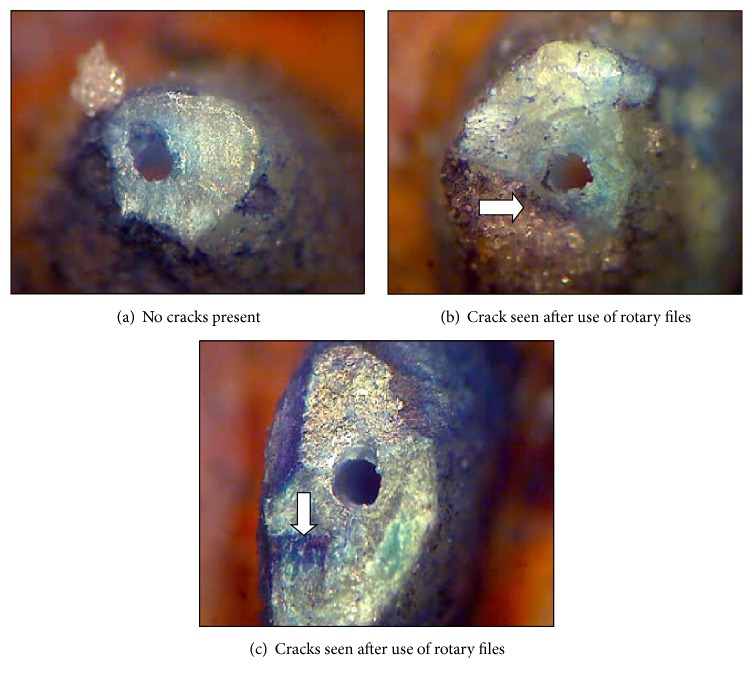
Images of dentinal cracks using different rotary file system.

In the present study, the ProTaper Universal finishing files were used to prepare the apical portion of the canals. The large apical taper of finishing files of this system generates increased stress on the dentinal walls as compared to the other rotary system which may increase the incidence of dentinal cracks [8] (see Figure 6). In this present study, when instrumentation with PTU and PTN was terminated at AF, AF + 1 mm, and AF − 1 mm, cracks were developed due to increase in torque with penetration depth of instrumentation and sharp apical curvature was located within the apical 2 mm. The reason for less number of cracks in PTN files instruments is due to its off-centered rectangular design which generates a swaggering motion, which decreases the screw effect, dangerous taper lock, and torque on any given file by minimizing contact between the file and dentin [9]. In addition, the PTN files are made of M-wire alloy which shows more flexibility than those made from conventional NiTi wire. The results obtained in the present study showed that dentinal cracks caused by PTU are not statistically significant compared to PTN. The mean percentage of dentinal cracks of PTU is 50% and PTN is 47%. The results are supported by study done by Lui et al. [10].

In the present study, when instrumentation with PTU and HyFlex CM was terminated at AF, AF + 1 mm, and AF − 1 mm, cracks developed. The reason for more cracks in PTU file is the more amount of material in core design and more percentage of increasing and decreasing taper design on single file, strategically less flexible than HyFlex CM. The results obtained in the present study showed that dentinal cracks caused by PTU are statistically significant compared to HyFlex CM. The mean percentage of dentinal cracks of PTU is 50% and HyFlex CM is 2.9%. The result obtained in the present study showed that dentinal cracks caused by using PTU are greater than HyFlex CM. These results are supported by study done by Capar et al. [11].

In the present study, when instrumentation with PTN and HyFlex CM was terminated at different instrumentation length at AF, AF − 1 mm, and AF + 1 mm, cracks developed. The reason for the less number of cracks in HyFlex CM is due to its 300% more resistance to cyclic fatigue. This control memory metallurgy of HyFlex CM file makes it more flexible but without the shape memory. This gives the file the ability to follow the anatomy of canal very closely reducing the risk of ledging, transportation, or perforation [12]. The results obtained in the present study showed more number of dentinal cracks at AF by PTN (31.3%) when compared to HyFlex CM (100%) at AF and no cracks were evident at AF + 1 mm and AF − 1 mm. The results obtained were supported by study done by Arslan et al. [13]. Alfoqom Alazemi et al. stated that the taper of files could be a leading factor in dentinal crack formation [14].

Thompson claimed that the tip design of rotary instruments, cross-sectional geometry, constant or variable pitch, and taper design and flute form could be related to crack formation [15]. All the instruments used in present study have noncutting tips and a variable pitch. In addition, both PTU and PTN instruments have a variable taper design, whereas the design of HyFlex CM is constant. Thus, in this present study the off-centered rectangular design of PTN instruments and relatively the high flexibility of both HyFlex and PTN instruments may have contributed to smaller number of cracks when compared to PTU instruments. The files used in present study had varying recommended speed and torque values.

The use of different speed and torque settings for each file system was a limitation of the present study. Recent study done by Peters et al. (2014) claimed that increased rotational speed was associated with increased cutting efficiency [16]. The second limitation of the present study was the difficulty in standardizing the force used during instrumentation.

## 7. Conclusion

Within the limitation of the present ex vivo study, none of the rotary files used in the present study showed dentinal cracks at AF − 2 mm when compared to the instrumentation at AF, AF − 1 mm, and AF + 1 mm. ProTaper Next and HyFlex CM rotary files have tendency to cause fewer dentinal cracks when compared with ProTaper Universal file system which showed the highest number of dentinal cracks.

## Figures and Tables

**Table 1:** Incidence of apical root cracks after canal preparation with rotary files at different instrumentation length.

File type	Number of cracks (*n* = 20)	Instrumentation length			
		AF + 1 mm (*n* = 5)	AF + 0 mm (*n* = 5)	AF − 1 mm (*n* = 5)	AF − 2 mm (*n* = 5)
PTN (*n* = 20)	16	8 (50.0)	5 (31.3)	3 (18.8)	0 (0.0)
PTU (*n* = 20)	17	8 (47.1)	7 (41.2)	2 (11.8)	0 (0.0)
HyFlex CM (*n* = 20)	1	0 (0.0)	1 (2.9)	0 (0.0)	0 (0.0)
Control (*n* = 5)	0	0 (0.0)	0 (0.0)	0 (0.0)	0 (0.0)
Total (*n* = 65)	34	16	13	5	0

**Table 2:** Comparison of incidence of cracks at instrumentation length between PTU and PTN groups.

File type	Number of cracks	Instrumentation length			*χ* ^2^ value	*p* value
		AF + 1 mm	AF	AF − 1 mm		
PTN	16	8 (50.0)	5 (31.3)	3 (18.8)	0.50	0.777
PTU	17	8 (47.1)	7 (41.2)	2 (11.8)		

**Table 3:** Comparison of incidence of cracks at instrumentation length between PTN and HyFlex CM groups.

File type	Number of cracks	Instrumentation length			*χ* ^2^ value	*p* value
		AF + 1 mm	AF	AF − 1 mm		
PTN	16	8 (50.0)	5 (31.3)	3 (18.8)	1.95	0.378
HyFlex CM	1	0 (0.0)	1 (2.9)	0 (0.0)		

**Table 4:** Comparison of incidence of cracks at instrumentation length between PTU and HyFlex CM groups.

File type	Number of cracks	Instrumentation length			*χ* ^2^ value	*p* value
		AF + 1 mm	AF	AF − 1 mm		
PTU	17	8 (47.1)	7 (41.2)	2 (11.8)	1.32	0.516
HyFlex CM	1	0 (0.0)	1 (2.9)	0 (0.0)		

**Table 5:** Comparison of incidence of cracks at instrumentation length between three groups.

File type	Number of cracks	Instrumentation length			*χ* ^2^ value	*p* value
		AF + 1 mm	AF	AF − 1 mm		
PTN	16	8 (50.0)	5 (31.3)	3 (18.8)	2.16	0.705
PTU	17	8 (47.1)	7 (41.2)	2 (11.8)		
HyFlex CM	1	0 (0.0)	1 (2.9)	0 (0.0)		

## Additional Points

The instrumentation of root canal alone significantly weakens the roots. Stresses are generated from inside of root canal which are higher in apical region and more along the walls, so the distribution of stress in apical area leads to development of cracks which may lead to root fracture. Clinically bacteria may proliferate in crack lines and later establish biofilms on the root surface.
